# Motivational interviewing for weight management among college students during COVID-19: An exploratory randomized controlled trial

**DOI:** 10.1016/j.obpill.2023.100097

**Published:** 2023-12-26

**Authors:** Kameron B. Suire, Jan Kavookjian, Kamden Strunk, Danielle D. Wadsworth

**Affiliations:** a Motivating Movement Lab, Kinesiology Department, Berry College, Mt. Berry, Georgia, USA; b Exercise Adherence and Obesity Prevention Laboratory, School of Kinesiology, Auburn University, Auburn, AL, USA; c Health Outcomes Research and Policy, Harrison School of Pharmacy, Auburn University, Auburn, AL, USA; d Foundations of Education, School of Education, Virginia Commonwealth University, Richmond, VA, USA

**Keywords:** Motivational interviewing, Obesity, Covid-19, College students, Body composition

## Abstract

**Background:**

College students encounter challenges in managing their weight. The Coronavirus Disease 2019 (COVID-19) pandemic exacerbated the problem. The purpose of this study was to determine the effect of a motivational interviewing (MI) intervention compared to online education (control) on body composition and self-determination theory constructs among college students with overweight.

**Methods:**

This was a randomized clinical trial of 40 college students comparing an MI versus a control group. The MI group received monthly interviews: three face-to-face interviews before the pandemic, and three video chat interviews after the outbreak of COVID-19 spanning a total of six months. The control group received six, monthly education modules. Body composition was measured by the iDexa and self-determination theory (SDT) variables were assessed with surveys.

**Results:**

Mixed ANOVAs from pre-post revealed significant changes in fat mass (p = .03, η^2^ = 0.22), lean mass (p < .05, η^2^ = 0.18), body fat percentage (*p* < .01, η^2^ = 0.37), autonomy (p < .01, η^2^ = 0.38), relatedness (p < .01, η^2^ = 0.41), amotivation (p = .01, η^2^ = 0.29), external regulation (p = .02, η^2^ = 0.23), identified regulation (p = .02, η^2^ = 0.25), integrated regulation (p < .00, η^2^ = 0.49), and intrinsic regulation (p = .01, η^2^ = 0.27).

**Conclusions:**

In this exploratory analysis, MI demonstrated a positive trend in body composition maintenance when compared to online education among overweight college students during a national pandemic. Future studies utilizing MI would enhance the literature by further investigating the relationship between MI and SDT and measuring body composition.

Clinicaltrials.gov. identifier: NCT04130386.

## Graphical abstract

**Figure:**
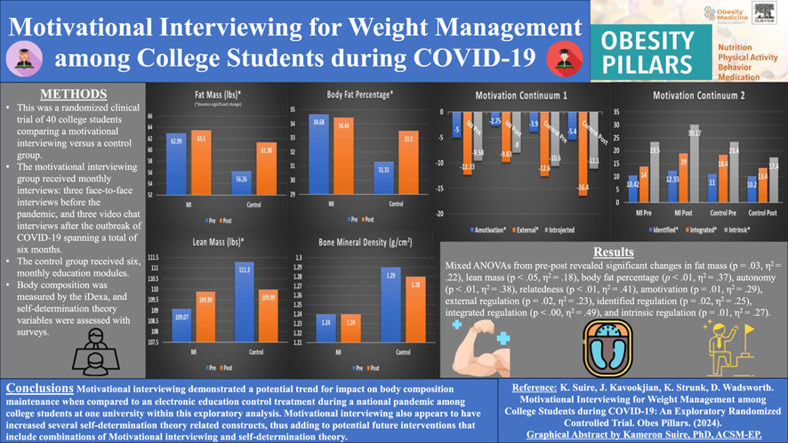

## 1 Introduction

Obesity is a consistent health concern among college students in the United States. According to the most recent national survey, 38.7 % of undergraduates have overweight or obesity according to self-reported height and weight [1]. Furthermore, comparing data from 2003 to 2004 and 2013–2014, shows childhood obesity rates remained stable (17.1 %–18.5 %), while adults showed an increasing trend (32.2 %–39.6 %) [2]. Unfortunately, obesogenic behaviors (e.g. lack of physical activity or excessive caloric consumption) have been exacerbated during Coronavirus Disease 2019 (COVID-19) quarantine restrictions resulting in decreases in physical activity and increases in weight among all age groups [[3], [4], [5], [6]]. This is concerning as obesity is associated with an increased risk for cardiovascular disease and diabetes, which are two of the leading causes of death in the United States [7]. Since almost two-thirds of adults attend college by age 30 in the United States [8], college campuses may be an ideal setting for obesity prevention interventions.

Prior to the pandemic, previous literature had pointed to mixed results for weight management interventions among college students. A systematic review and meta-analysis showed that while 34 of the 41 retained (met criteria for inclusion in the review/meta-analysis) studies reported significant improvements in at least one outcome, the overall base of literature has not demonstrated adequate success [9]. For the physical activity outcomes in the meta-analysis retained studies, no significant changes in total and vigorous physical activity were reported in intervention groups compared to control groups. While there were significant changes in moderate physical activity, the effect was small (SMD = 0.18). Half of retained study interventions that included nutritional outcomes showed statistical significance, with most centering on fruit and vegetable consumption. Finally, only 3 of 11 interventions demonstrated statistically significant changes in weight related outcomes. It is important to mention that only half of the total reviewed interventions were randomized controlled trials (RCT)s, and studies relied on weight and body mass index (BMI) for anthropometric status rather than body composition measures. These gaps in the literature highlight the need for robust and impactful studies to further advance behavioral adherence related to weight gain.

Motivational interviewing (MI) is an empathetic and person-centered communication approach [10] that has demonstrated success in increasing adherence to various health behaviors [[11], [12], [13]], including weight management for children with overweight [14], adults with overweight in primary care [15] and women with overweight [16]. Specifically in the field of obesity medicine, MI is a cooperative discussion between health professionals and patient/participant intended to promote healthy behaviors for weight management [17]. A meta-analysis employed for weight management among patients with overweight found an effect size of 0.51 (95 % CI -1.04, 0.01; *p* = .05) for weight with 12 included studies [15]. Another meta-analysis conducted among women found an effect size of 0.19 (95 % CI -0.13, 0.26; *p* < .01) for weight with 8 studies included, and the effect size of 0.35 (95 % CI -0.12, 0.58; *p* < .01) for BMI with 6 studies included [16]. However, there is very little research on MI-based weight management interventions for college students.

The original objective of this study was to examine the effectiveness of MI on physiological outcomes after a three-month long intervention. However, as the pandemic emerged across the country, our study protocol was altered (extension to six months) and the adapted intervention offers a unique perspective into how MI can impact weight management across environments and during a pandemic that included social distancing and quarantine. Therefore, the updated objective of the intervention was to examine the effectiveness of MI on physiological outcomes after a six-month long intervention, with the last three months of MI being conducted remotely. When COVID-19 restrictions were enacted, most adults were confined to stay at home for varying lengths during the pandemic [18]. Universities and colleges across the country were closed or converted to online/virtual learning only, resulting in many college students losing their current homes and having to move back home to their prior living arrangements. This disruption had a substantial impact on various health behaviors including those that related to weight management among college students. Increased junk food eating, binge eating, food insecurity, and less vegetable consumption were consistent findings [19]. Decreased physical activity and increased sedentary behaviors also increased during this time period [20,21]. College students also showed increases in weight [19,22,23].

In addition to investigating physiological impact, a second objective of this research was to explore the potential impact of MI on psychosocial factors derived from self-determination theory (SDT) related constructs. It has been theorized that MI may have a relationship with SDT by integral figures from both fields [[24], [25], [26]]. More specifically, Markland and colleagues [24] suggest that MI may provide an environment in which the three psychological needs of autonomy, competence, and relatedness are satisfied, and therefore, more self-determined behavior may occur. There are currently few studies that measure SDT related constructs after an MI intervention and the theory-based investigation is warranted.

## 2 Methods

This RCT was approved by the University Institutional Review Board for Research Involving Human Subjects (IRB) and followed the standards set by the Declaration of Helsinki; the registered clinical trial number is NCT04130386. Each participant read and signed a written informed consent and completed the Physical Activity Readiness Questionnaire (PAR-Q). Participants had to answer “no” to all questions on the PAR-Q to participate in the intervention.

College students were recruited by word of mouth, e-mail, flyers and social network blast within the university community in advance with a three-week window to join the study. To be eligible for this study, participants had to be: a college student, low risk for medical complications from exercise (as determined by the PAR-Q), currently not exercising, over 25 BMI, and not pregnant. Participants who qualified for the study based on these requirements were randomized to either the MI group or the online education (control) group. Randomization occurred by a flip of a coin by a research assistant with heads resulting in randomization into the MI group and tails resulting in randomization into the control group. The same research assistant enrolled the participant and allocated them to the respective group after the coinflip. All measures were assessed prior to randomization.

### 2.1 MI group

The intervention group received three in-person MI sessions and three video chat MI sessions all lasting 30 min over the course of six months. These MI sessions were delivered monthly. These in-person MI sessions were conducted on campus at the testing location, meaning participants were required to travel to the testing location. Originally, only three MI sessions were planned but the last three video chat sessions were added as the study setting transitioned to COVID-19 restrictions and the participants couldn't return to campus for in-person encounters or post-testing. These restrictions that prevented post-testing resulted in our team extending the study by three months, leading to a six-month intervention. Aside from the three-month extension and the addition of three video chat MI sessions for the intervention group and three more educational emails for the control group, all other outcomes and protocols remained the same. The MI sessions were conducted by one trained exercise physiologist. Sessions were rooted in the spirt of MI approach with a focus on eliciting change talk and goal setting for weight management behaviors while remaining person-centered. Topics ranged from physical activity, nutrition, stress management, alcohol consumption, and sleep. It is important to note that since the sessions were person-centered, the interviews revolved around the subject's concerns, motivations, life routine, and goals.

### 2.2 Training and fidelity

Because MI is complex and person-centered, adequately training the interventionist in MI and conducting intervention fidelity assessment of the intervention is standard practice [27]. The interviewer in this intervention underwent an extensive, evidence-based 16-week training with significant conceptual development before engaging in skills application exercises 12 months prior to this intervention. The training involved learning the origins and philosophy of MI, watching and critiquing example videos, written dialog exercises, and extensive group-based role-play with MI-expert feedback, along with coaching from peers and an MI expert. Additional follow-up MI exposures were gained over the next 12 months leading up to this intervention through four supervised 4-h experiences facilitating the role play and feedback processes in MI trainings for others. Feedback was given throughout these additional follow-up exposures leading to further growth of the MI deliverer.

Fidelity measures post-training and during the intervention were employed to support claims for MI-adherent implementation. To assess MI fidelity post-training, an 8-min simulated encounter with a trained standardized patient was conducted. In addition, at the start of the study, the first five interviews were also coded, and direct feedback and coaching was then provided to the interventionist to identify strengths and areas for improvement. During the intervention, all participant encounters were audio recorded and about 30 % were randomly selected (stratified to three sessions at each of the six study time points equaling 18 sessions total) for fidelity assessment by a trained, MI expert using the Motivational Interviewing Treatment Integrity Code (MITI) 4.1.2 [28]. 20 min of these interviews were randomly selected for coding. The MITI 4.2.1 is a behavioral coding system used to monitor fidelity to MI and has two components: global scores and behavior counts. A global score entails the coder to assign a single number from a five-point scale to characterize an entire interaction. Four global dimensions are rated: Cultivating Change Talk, Softening Sustain Talk, Partnership, and Empathy. A behavior count requires the coder to tally instances of interviewer behaviors. The various behavior counts included: giving information, persuading, persuading with information, question, simple reflection, complex reflection, affirm, seeking collaboration, emphasizing autonomy, and confront.

### 2.3 Control group

The control group consisted of monthly education materials sent via email for six months. Topics covered in the first three months included: physical activity, nutrition, stress management, managing alcohol intake, and time management. After the onset of restrictions related to the COVID-19 pandemic (final three months), educational material centered on various forms of physical activity that could be performed at home. Educational content delivered via email was selected due to its typical usage in RCT MI interventions for weight management [15]. Usual care is one of the most popular forms of control groups within weight management interventions utilizing MI, which typically is educational content. Print materials or leaflets are usually the form that this educational content takes, though since every student has an active student email, we decided to deliver the content utilizing email. This content was delivered to match the dosage within the MI group as a once per month intervention.

### 2.4 Body composition measures

The iDexa measured body composition on participants in a fasted state (no nutritional intake for the prior 8 h) by trained personnel. iDexa measures body composition and bone density by dual energy X-ray absorptiometry which provides accurate data related to body composition in terms of BMI, body fat, lean mass, bone mineral density (BMD) and exact data from sections of the body if necessary [29,30]. Participants are instructed to lay still on the iDexa machine while the X-ray beam energy passes over the entire body, a process that takes between 7 and 14 min depending on the thickness of the body. Specifically, variables of interest for this study include lean mass (kg), fat mass (kg), body fat percentage, and BMD (g/cm^2^). According to previous studies, the precision error for total body mass was 0.9 %, total body lean mass was 0.4–0.5 %, total bone mineral content was 0.6 %, fat mass was 0.7–0.8 %, and percent body fat was 0.6–0.9 %, which are all considered to be excellent [[31], [32], [33]].

### 2.5 SDT measures

Questions regarding demographics resided in the base questionnaire and included race, sex as assigned at birth, and age. Psychological constructs falling under the realm of SDT included behavioral regulation, autonomy, relatedness and competence were completed pre and post. The Behavioral Regulation Exercise Questionnaire version 3 (BREQ-3) is a 24 item 5-point (ranging from “not true for me” to “very true for me”) Likert -type scale used to detail where participants fall on the continuum of behavioral regulation [34]. The questionnaire consists of 24 items with 4 questions for each subscale (amotivation, external, introjected, identified, integrated, and intrinsic). Amotivation describes a missing intention for action or simply acting aimlessly. External regulation occurs when actions revolve solely around external rewards or consequences. Introjected regulation occurs when actions revolve around guilt, pressure from important people, and motivation to conform to social norms. Identified regulation occurs when the action is personally valued due to its involvement with a personal goal. Integrated motivation occurs when actions are directed by an integrated form of identity built of values that have become a part of the self. Intrinsic regulation which occurs when the actions are guided by enjoyment, interest, or knowledge and the action is valued for itself rather than any consequences because of the action [35]. Each subscale was differentially weighted: amotivation, −3; external regulation, −2; introjected regulation, −1; integrated regulation +1; identified regulation, +2; intrinsic regulation, +3. Scores for amotivation can range from −60 to 0. Scores for external regulation can range from-40 to 0. Scores for introjected regulation can range from −20 to 0. Scores for integrated regulation can range from 0 to 20. Scores for identified regulation can range from 0 to 40. Scores for intrinsic regulation can range from 0 to 60. Previous researchers provided evidence of content and criterion validity, and found strong score reliability [34,36,37]. In the current study, reliability scores of the scales within the BREQ-3 ranged from α = 0.76 to 0.97.

The Basic Psychological Needs Exercise Scale (BPNES) is a 5-point (ranging from “I don't agree at all” to “I completely agree”) Likert-type scale which measures autonomy and competence satisfied in exercise [38] scale includes 8 items, 4 each representing autonomy and competence. Scores for both autonomy and competence can range from 4 to 20. The need of competence is the understanding that humans need to have mastery over their environment and feel adequate and competent. The need for autonomy refers to a need for control in one's ventures and an internal locus of causality [39]. Previous researchers provided evidence of content and criterion validity, and found strong score reliability [38,40]. Within the current study, the reliability scores of autonomy and competence were α = 0.96 and 0.97 respectively.

The Relatedness to Others in Physical Activity Scale (ROPAS) is a 6-point (ranging from “false” to “true”) Likert-type scale which assesses the psychological need of relatedness, specifically focusing on physical activity [37]. The need for relatedness refers to a need to feel connected and a sense of belonging [39]. The scores for relatedness can range from 6 to 36. Previous researchers provided evidence of structural and criterion validity and found strong score reliability [30]. In the present study, the reliability score for the ROPAS scale was α = 0.99. While the BPNES scale also measures relatedness, it focuses on structured exercise exclusively and with whom the participant exercises; however, relatedness experienced in exercise may be served in other ways. For example, the ROPAS scale inquires “I am supported by others in this activity” and “I have a close bond with others”. These questions do not rely on an exercise partner as relatedness can be fulfilled by those not physically exercising with the participant.

### 2.6 Data analysis

Mixed ANOVAs assessed for statistically significant differences in variables. The mixed ANOVA allows for testing at the interaction of a between-subjects factor (MI versus control group in the present study) and a within-subjects factor (pre- and post-test in the present study). Although the sample size under analysis was smaller than is typically expected in an ANOVA design, it was still the appropriate choice as the model's statistical assumptions were met [41,42]. G∗power indicated a required sample size of 68 [43]. Probability values of *p* < .05 were considered significant.

## 3 Results

### 3.1 Participants

**Fig. 1:**
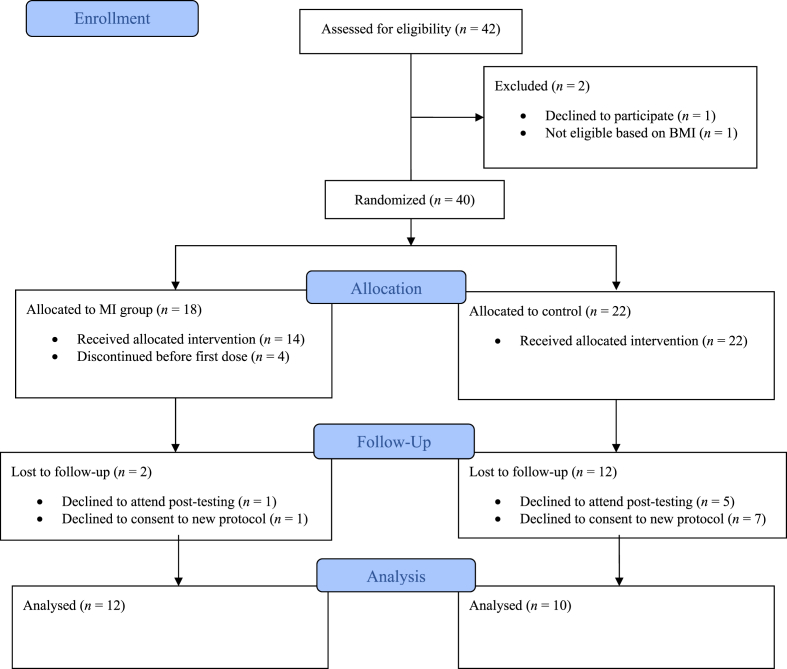
A CONSORT 2010 flow diagram.

A total of 40 college students were randomly assigned to groups. Information describing participants can be found in Table 1. Demographic characteristics and BMI were not statistically significantly different at baseline between experimental conditions. See Fig. 1 for the trial flow diagram of participant recruitment, participation protocol, and attrition. Attrition was much larger than expected compared with weight loss-based MI interventions reported in a meta-analysis at six months [15]. The larger attrition rate in this study appeared to be related to complications arising from the COVID-19 pandemic. Four of the 18 participants who had been randomized to the intervention group dropped out of the intervention before the first session of MI. Eight participants declined to sign the updated consent form with the approved extension due to the pandemic. Another six participants declined to attend the post-testing appointment. The major reasons participants gave for declining to continue with the study were issues with the extended protocol (moving/graduation, lack of time, etc.) and lack of ability to attend post-testing protocol during the pandemic. The latter reason often included participants that no longer lived in the area due to an exclusive online schedule for the fall semester. The final sample size included 12 participants in the intervention and 10 in the control group.

**Table 1:** Means (standard deviations) and percentages for demographic variables at baseline.

Variable	MI (*n* = 18)	EE (*n* = 22)	Total (*n* = 40)
Age (yrs)	20.87 (1.92)	21.24 (2.88)	21.70 (2.47)
BMI kg/m^2^	30.28 (6.28)	27.92 (3.31)	28.99 (4.95)
% Female	66.7 %	81.8 %	75.0 %
% Male	33.3 %	18.2 %	25.0 %
% White	88.9 %	77.3 %	82.5 %
% Black	5.6 %	18.2 %	12.5 %
% Hispanic	5.6 %	4.5 %	5.0 %

MI=Motivational Interviewing, EE = Electronic Education, BMI=Body Mass Index.

### 3.2 Body composition

Table 2 displays mean scores for pre- and post-testing variables by treatment condition. A significant interaction was noted for fat mass *F* (1, 20) = 5.52, *p* = .03, η^2^ = 0.22 by group by time. The MI group gained fat mass (*M* = 0.23 kg, *SD* = 1.90) compared to the much larger gain within the control group of (*M* = 2.32 kg, *SD* = 2.28). A significant interaction for group by time was also noted for lean mass, *F* (1, 20) = 4.51, *p* < .05, η^2^ = 0.18. The MI group gained lean mass (*M* = 0.37 kg, *SD* = 1.20) compared to the loss of (*M* = −0.59 kg, *SD* = 0.86) within the control group. There was also a significant interaction discovered for body fat percentage (*F* (1, 20) = 11.85, *p* < .01, η^2^ = 0.37. The MI group decreased body fat percentage (*M* = −0.24 %, *SD* = 1.48) compared to a gain in the control group (*M* = 2.19 %, *SD* = 1.84). There was no signiﬁcant interaction found with BMD, *F* (1, 20) = 2.44, *p* = .13, η^2^ = 0.11. Observed power for group by time interactions ranged from 0.32 to 0.91, indicating less power than the recommended 0.80 in all but one measure of body composition, which was body fat percentage with a power of .91.

**Table 2:** Mean scores (standard deviations) by treatment condition.

Variable	MI Pre	MI Post	EE Pre	EE Post	*p*	*Effect size (*$\eta_{p}^{2}$*)*
Body Composition						
Fat Mass (kg)	28.57 (16.41)	28.80 (16.77)	25.52 (12.97)	27.84 (13.53)	0.03∗	.22
Body Fat Percentage	34.68 (11.58)	34.44 (12.02)	31.31 (9.13)	33.50 (8.68)	0.00∗∗	.37
Lean Mass (kg)	49.47 (8.39)	49.84 (8.29)	50.48 (11.58)	49.89 (11.35)	0.05∗	.18
BMD	1.24 (.13)	1.24 (.14)	1.29 (.10)	1.28 (.10)	0.13	.11
**Psychological Variables**						
Autonomy	12.58 (4.64)	15.08 (3.34)	15.20 (3.12)	11.40 (3.72)	0.00∗∗	.38
Competence	12.33 (4.19)	12.08 (3.00)	13.20 (3.26)	10.10 (3.35)	0.11	.13
Relatedness	24.42 (9.08)	26.00 (6.25)	26.30 (8.68)	20.20 (6.78)	0.00∗∗	.41
Amotivation	−5.00 (7.82)	−2.75 (5.35)	−3.90 (5.49)	−5.40 (7.59)	0.01∗	.29
External	−12.33 (8.73)	−9.83 (7.31)	−12.60 (9.57)	−16.40 (10.41)	0.0∗	.23
Introjected	−9.58 (3.50)	−8.00 (5.01)	−10.60 (2.22)	−11.10 (2.23)	0.08	.15
Identified	10.42 (2.68)	12.33 (2.31)	11.00 (2.94)	10.20 (2.78)	0.02∗	.25
Integrated	14.00 (7.19)	19.00 (5.49)	18.40 (6.98)	13.40 (3.13)	0.00∗∗	.49
Intrinsic	23.50 (12.78)	30.17 (10.95)	23.40 (11.47)	17.40 (11.03)	0.01∗	.27

MI=Motivational Interviewing, EE ​= ​Electronic Education, ∗Denotes a significant change (p ​< ​.05). ∗∗ Denotes value less than 0.01.

### 3.3 SDT constructs

Statistically signiﬁcant interactions for group by time were found for several variables. A significant effect between groups was noted for autonomy, *F* (1, 20) = 12.28, *p* < .01, η^2^ = 0.38. The MI group reported an increase in mean autonomy (*M* = 2.50, *SD* = 3.70) as compared to the decrease (*M* = −3.80, *SD* = 4.78) in the control group. A significant effect between groups was found for relatedness, *F* (1, 20) = 13.77, *p* < .01, η^2^ = 0.41. An increase in mean relatedness (*M* = 1.58, *SD* = 5.18) was found in the MI group when compared to the decrease (*M* = −6.10, *SD* = 4.38) in the control group. A significant effect between groups was found for amotivation, *F* (1, 20) = 8.22, *p* = .01, η^2^ = 0.29. The MI group had a significantly higher decrease in mean amotivation (*M* = −2.25, *SD* = 2.90) compared to the decrease (*M* = −0.90, *SD* = 3.48) in the control group. A significant effect between groups was noted for external regulation, *F* (1, 20) = 6.04, *p* = .02, η^2^ = 0.23. There was a decrease in the MI group regarding mean external regulation (*M* = −1.67, *SD* = 3.60) when compared to the increase (*M* = 3.00, *SD* = 7.56) in the control group. A significant effect between groups was found for identified regulation, *F* (1, 20) = 6.79, *p* = .02, η^2^ = 0.25. The MI group had an increase in mean identified regulation (*M* = 1.92, *SD* = 2.54) compared to the decrease (*M* = −0.80, *SD* = 2.30) in the control group. A significant effect between groups was also noted for integrated regulation, *F* (1, 20) = 19.27, *p* < .00, η^2^ = 0.49. There was an increase for mean integrated regulation (*M* = 5.00, *SD* = 5.63) within the MI group compared to the decrease (*M* = −5.00, *SD* = 4.92) in the control group. Finally, a significant effect between groups was found for intrinsic regulation, *F* (1, 20) = 7.26, *p* = .01, η^2^ = 0.27. The MI group had an increase (*M* = 6.67, *SD* = 7.88) in mean intrinsic regulation compared to the decrease (*M* = −6.00, *SD* = 13.86) in the control group. No signiﬁcant change across groups was found for competence *F* (1, 20) = 2.85, *p* = .11, and introjected regulation *F* (1, 20) = 3.45, *p* = .08. Table 2 provides results for all psychological variables. Observed power for group by time for psychological variables ranged from 0.36 to 0.99, with competence, amotivation, external regulation, introjected regulation, identified regulation, and intrinsic regulation having less than 0.80 power.

### 3.4 MI fidelity

Table 3 presents fidelity to MI as measured by the MITI 4.2.1. Summary scores were utilized to compile the results from all interviews in a more concise manner rather than detailing each individual interview [28]. Relational scores are the sum of both partnership and empathy global scores. Technical scores are the sum of cultivating change talk and sustaining change talk global scores. Percentage of complex reflections (%CR) is the total percentage of behavior counts for reflections that are complex as opposed to simple. Reflection-to-question ratio (R:Q) is the ratio of behavior counts of reflections compared to questions. Total MI-adherent (MIA) is the total sum of behavioral counts of seeking collaboration, affirm, emphasizing autonomy. Total MI Non-Adherent (MINA) is the sum of behavioral counts of confront and persuade. Interventionists exceeded “fair” proficiency thresholds for relational, technical, and %CR. The only category that the intervention fell below fair proficiency was the R:Q category. No recommended proficiencies are present for MIA and MINA.

**Table 3:** Motivational interviewing proficiency compared with recommended proficiencies using the MITI 4.2.1

MITI Domain	Mean	Fair	Good
Relational	4.36	3.5	4
Technical	4.39	3	4
%CR	47.5 %	40 %	50 %
R:Q	.93:1	1:1	2:1
MIA	14.95		
MINA	0.17		

%CR=Complex reflections, R:Q = Reflection-to-question ratio, MIA = Total MI-adherent, MINA = Total MI Non-Adherent.

## 4 Discussion

The purpose of this study was to determine the effect of a MI intervention on body composition and SDT variables among college students with overweight or obesity. As most of this study occurred during the COVID-19 pandemic, this study offers insight into how MI effects are sustainable in varying environments and delivery modes.

### 4.1 College weight management literature

When looking at the literature for weight loss inventions among college students, a previous systematic review showed only 3 of 11 interventions reported significant anthropometric changes among college students [9]. These interventions relied on weight or BMI as the measure of anthropometric outcomes, although since this review was completed, two interventions have emerged that utilized more advanced anthropometric measures. Mustedanagic’ and team [44] utilized skinfolds to measure body composition and found that after 36 aerobic and resistance training sessions over 12 weeks lasting 60 min, there was a significant decrease in body fat percentage and a significant increase in lean mass percentage. Another study utilized biological impendence to measure body fat percentage and skeletal muscle after a very intensive intervention of 60 aerobic and 60 resistance training sessions over 12 weeks and found significant decreases in body fat percentage and a significant increase in skeletal muscle pre to post [45].

The current study used six, 30-min interviews over six months, which is a much less intensive intervention comparatively, and found a difference of 2.43 % in body fat between the two groups at the end of the study, which is similar to the difference of 2.5 % reported by Mustedanagic et al. [44], though less than the 5.5 % loss from pre to post among the intervention conducted by Kim & Han [45]. Most of the interventions addressing weight status among college students also appear to be short-term interventions (10–12 weeks) versus the six-month intervention in this study. Future interventions are needed among college students that demonstrate impact on anthropometric status. Interventions that utilize follow-up periods and measure body composition would make beneficial contributions to the current literature.

### 4.2 MI weight management literature

Two meta-analyses have measured anthropometric changes of MI interventions. Armstrong et al. [15], found that in MI RCT's patients with obesity or overweight in the MI groups lost about 1.47 kg and 0.25kg/m2 BMI more than control groups. Suire and team [16] found that among adult women, MI RCT's reported that participants receiving the MI intervention lost about 1.36 kg and −1.22kg/m2 in BMI more than control groups. The intervention in the current study demonstrated comparable results with the MI group showing about 1.80 kg and 0.70 kg/m2 less compared to the control. These results demonstrate similar effectiveness to that reported in the literature, even during restrictions from a pandemic, though it is vital to mention that our low sample size should be considered. As most MI interventions measuring anthropometric outcomes rely on weight or BMI [[14], [15], [16]], this study provides further information in body composition changes. While useful and often more feasible, weight and BMI measures give very little information about effects of the intervention on actual body composition. It is important to note that in this study there were significant differences between MI and online education groups for both fat mass and lean mass, in addition to a non-significant, yet positive trend for BMD. These differences would not have been detected by relying on weight or BMI and may signal a need for future studies to incorporate more objective measures of body composition. This would propel the literature forward and provide health professionals with vital information due to the link between fat mass and lean mass with mortality [46].

This research acted upon gaps in the literature identified in previous research [15,16] which was the lack of training and fidelity information. This information is imperative to establish future standards for ensuring that MI was delivered at an adherence threshold determined to be effective by MI experts. Detailed training and fidelity procedures were reported within this research report and demonstrated consistent fidelity across the study encounters, even after the transition from in-person to virtual nature of the encounters. As per fidelity scores, MI-adherence was delivered during the intervention, supporting claims for validity that MI was the core component of this intervention that impacted the outcomes as described. Future interventions should report both detailed training information and fidelity results to strengthen MI interventions and provide a base of evidence.

### 4.3 SDT-MI literature

Literature assessing SDT-related constructs in MI weight management interventions are still in the early stages. One prior intervention reported decreases in participant-reported amotivation while showing increases in identified and integrated regulation [47]. Another study found that an intervention combining principles from SDT and MI had higher psychological basic need support when compared to a control group, and that perceived competence mediated results [48]. In addition, two interventions demonstrated MI participants had increases in autonomous motivation as well as weight loss [49,50]. Webber and collaborators [49] assessed autonomous motivation in an 8-week internet-based intervention comparing MI to MI plus a discussion of values. Autonomous motivation increased in both groups and higher autonomous motivation at follow-up was associated with greater weight loss. It also was found that more change talk during the MI sessions was correlated with a higher autonomous motivation. West and team [50] directly mentioned SDT as a part of a theoretical framework for a motivation-focused approach which included an autonomy-driven component based on MI. This was compared to a skills-based approach as well as a control group for effect on weight loss maintenance among women with overweight at follow-up after a six-month weight loss intervention. Both the motivation (−5.34 kg) and skills-focused (−5.22 kg) groups lost a statistically significant amount of weight compared to the control, while not being significantly different from each other. Participants in the motivational group had significantly higher autonomous motivation for weight control than the skills-based group at the mid-point of the follow-up time point. Taking these findings into consideration along with our findings that demonstrate significant increases in the psychological needs of autonomy and relatedness in addition to identified motivation, integrated motivation, and intrinsic motivation when compared to the control group, future researchers may benefit from measuring SDT related constructs in MI interventions. These findings could prove beneficial due to the literature demonstrating a relationship with SDT constructs and weight loss and related behaviors [[51], [52], [53]].

### 4.4 COVID-19 literature

Evidence has shown consistent evidence for weight gain during the onset COVID-19 pandemic [19]. The vast majority of the literature focuses on self-reported data on whether their sample gained weight or not. There were instances of 18 % [54], 19 % [55], 27 [56]%, 32 [57]%, 36 [58]%, 37 [59]%, 53 [60]%, 57 [61]%, and 64 [62]% of the student samples gaining weight months after the pandemic began. Though not apart of our main outcomes, utilizing our data, we found that about 73 % of our sample gained weight indicating a higher amount of weight gain compared to the literature. Within the MI group, 58 % gained weight compared to 90 % within the control group. One intervention reported specific weight data and found a modest increase from 67.4 ± 14.9 kg to 68.0 ± 15.2 kg [63]. Once again, though not apart of our main outcomes, our study found an increase in weight from 83.28 kg ± 17.76–84.57 kg ± 17.14 for a total of 1.29 kg. The MI group gained .47 kg compared to the 2.27 kg gained by the control group. The MI group had a slightly smaller weight gain whereas the control gained a larger amount of weight when compared to the intervention by Brancaccio and team [63]. It is important to note the lack of body composition data among college students during the onset of the pandemic. While weight data is certainly useful, it doesn't provide the entire story. In the current study, the control group showed college students with overweight gained an average of 2.33 kg of fat mass during a six-month span (most of which was during the onset of the pandemic). The control group also lost .59 kg of lean mass, a curious finding that is novel within the literature. Not only does this potentially indicate a gain in fat mass due to a lack of physical activity, but also a loss of muscle mass. The MI group was able to maintain their fat mass and lean mass providing support that MI may be an effective intervention strategy to prevent fatness gain in limited access environments.

### 4.5 Limitations

Steps were taken to minimize threats to validity in the study design, but all limitations should be noted. Perhaps the most significant limitation of this study was the small sample size. While small sample sizes are not uncommon in this type of research, this resulted in low statistical power which could have impacted the ability to detect difference between the intervention and control groups. The post-test sample size was much smaller than the suggested power analysis sample size of 68 from G∗power. Related to this challenge was the unusually high attrition rate. With the COVID-19 pandemic spreading in the middle of this intervention, many participants dropped out of the study for a variety of reasons including graduation, not returning to campus or passively withdrawing from the intervention. In addition, generalizability of the findings may be limited due to not only these challenges but that the study was conducted at one university among a mostly white, female sample. Future interventions would greatly benefit the literature by increasing diversity among samples. There was also the limitation of being a per-protocol analysis rather than intention to treat, which may introduce bias. Another limitation was the lack of blinding of the researchers to the group assignment of participants. Blinding cannot truly occur with a MI intervention due to the personal interaction between the subject and MI deliverer. It is also important to mention a lack of detail within this intervention on dosage of the MI sessions in addition to engagement with the electronic educational material among the control group. It is possible that the participants within the control group didn't engage with the content at a high level, thus making it a poor choice for a control group. Future interventions would enhance the literature by providing this information. In addition, participants may have responded to self-reported surveys of SDT and behavior adherence with social desirability bias and/or recall bias. The next limitation is that there was only one interviewer that delivered MI, which may have introduced bias. Although this may be mitigated by the fidelity assessments for MI delivery. Future interventions will likely need utilize multiple interviewers as the sample size increases. The final limitation was the lack of measurements on weight related behaviors, such as physical activity and nutrition throughout the study. Measuring these behaviors during the pandemic proved to be problematic and future studies would benefit from including them.

## 5 Conclusion

MI demonstrated a potential trend for impact on body composition maintenance when compared to an electronic education control treatment during a national pandemic among college students at one university within this exploratory analysis.

• Body composition data on college students right before and after the onset of the pandemic is novel and this intervention provides much needed information.
• MI also appears to have increased several SDT related constructs, thus adding to potential future interventions that include combinations of MI and SDT.
• Participants in the control group gained a substantial amount of fat mass while losing lean mass, potentially signaling additional health impacts from large shutdowns.

## Author contribution

Kameron Suire – overall design of the study, data collection, writing/revising of the manuscript.

Jan Kavookjian – Fidelity assessment, full text review, writing/revising of the manuscript.

Kamden Strunk- Statistical Analysis, full text review, writing/revising of the manuscript.

Danielle Wadsworth – overall design of the study, oversaw data collection and data integration, writing/revising of the manuscript.

## Ethical review

This randomized controlled trial (RCT) was approved by the University Institutional Review Board for Research Involving Human Subjects (IRB) and followed the standards set by the Declaration of Helsinki; the registered clinical trial number is NCT04130386. Each participant read and signed a written informed consent and completed the Physical Activity Readiness Questionnaire (PAR-Q). Participants had to answer “no” to all questions on the PAR-Q to participate in the intervention.

## Funding

No outside research grant funding was used to conduct this study. The research team were employees at Auburn University when this study was conducted. Laboratory funds from the Exercise Adherence and Obesity Prevention Laboratory within the School of Kinesiology at Auburn University paid for additional costs of the study.

## Declaration of Artificial Intelligence (AI) and AI-assisted technologies

During the preparation of this work the authors did not use AI.

## Declaration of competing interest

Dr. Kavookjian reports that she is on the Merck Speakers Bureau for non-product medical education for the topics of Motivational Interviewing, Shared Decision-Making and Health Literacy Communication; Dr. Kavookjian also consults for Merck as the motivational interviewing content expert for patient-centered education materials; Dr. Kavookjian also consults for MediMergent, LLC for motivational interviewing training. All other authors state they have no conflicts of interest to report.

## References

[1] American College Health Association (2020).

[2] Hales C.M., Carroll M.D., Fryar C.D., Ogden C.L. (2017).

[3] Bhutani S., VanDellen M.R., Cooper J.A. (2021). Longitudinal weight gain and related risk behaviors during the COVID-19 pandemic in adults in the US. Nutrients.

[4] Chang T.H., Chen Y.C., Chen W.Y. (2021). Weight gain associated with COVID-19 lockdown in children and adolescents: a systematic review and meta-analysis. Nutrients.

[5] Chew H.S., Lopez V. (2021). Global impact of COVID-19 on weight and weight-related behaviors in the adult population: a scoping review. Int J Environ Res Publ Health.

[6] Anderson L.N., Yoshida‐Montezuma Y., Dewart N. (2023). Obesity and weight change during the COVID‐19 pandemic in children and adults: a systematic review and meta‐analysis. Obes Rev.

[7] Kochanek K.D., Murphy S.L., Xu J., Arias E. (2017).

[8] U.S. Census Bureau (2019).

[9] Plotnikoff R.C., Costigan S.A., Williams R.L. (2015).

[10] Miller W.R., Rollnick S. (2012).

[11] Burke B.L., Arkowitz H., Menchola M. (2003). The efficacy of motivational interviewing: a meta-analysis of controlled clinical trials. J Consult Clin Psychol.

[12] Lundahl B.W., Kunz C., Brownell C., Tollefson D., Burke B.L. (2010). A meta-analysis of motivational interviewing: twenty-five years of empirical studies. Res Soc Work Pract.

[13] Rubak S., Sandbæk A., Lauritzen T., Christensen B. (2005). Motivational interviewing: a systematic review and meta-analysis. Br J Gen Pract.

[14] Suire K.B., Kavookjian J., Wadsworth D.D. (2020). Motivational interviewing for overweight children: a systematic review. Pediatrics.

[15] Armstrong M., Mottershead T., Ronksley P., Sigal R., Campbell T., Hemmelgarn B. (2011). Motivational interviewing to improve weight loss in overweight and/or obese patients: a systematic review and meta-analysis of randomized controlled trials. Obes Rev.

[16] Suire K.B., Kavookjian J., Feiss R., Wadsworth D.D. (2020).

[17] Freshwater M., Christensen S., Oshman L., Bays H.E. (2022). Behavior, motivational interviewing, eating disorders, and obesity management technologies: an Obesity Medicine Association (OMA) Clinical Practice Statement (CPS) 2022. Obes Pillars.

[18] Bhutani S., Cooper J.A. (2020). COVID‐19 related home confinement in adults: weight gain risks and opportunities. Obesity.

[19] Jehi T., Khan R., Halawani R., Dos Santos H. (2023). Effect of COVID-19 outbreak on the diet, body weight and food security status of students of higher education: a systematic review. Br J Nutr.

[20] Wilson O.W., Holland K.E., Elliott L.D., Duffey M., Bopp M. (2021). The impact of the COVID-19 pandemic on US college students' physical activity and mental health. JPAH.

[21] Olfert M.D., Wattick R.A., Saurborn E.G., Hagedorn R.L. (2022). Impact of COVID-19 on college student diet quality and physical activity. Nutr Health.

[22] Dun Y., Ripley-Gonzalez J.W., Zhou (2021). Weight gain in Chinese youth during a 4-month COVID-19 lockdown: a retrospective observational study. BMJ Open.

[23] Chen H.W.J., Marzo R.R., Anton H. (2021). Dietary habits, shopping behavior and weight gain during COVID-19 pandemic lockdown among students in a private university in Selangor, Malaysia. J Public Health Res.

[24] Markland D., Ryan R.M., Tobin V.J., Rollnick S. (2005). Motivational interviewing and self–determination theory. J Soc Clin Psychol.

[25] Miller W.R., Rollnick S. (2012). Meeting in the middle: motivational interviewing and self-determination theory. IJBNPA.

[26] Vansteenkiste M., Sheldon K.M. (2006). There's nothing more practical than a good theory: integrating motivational interviewing and self-determination theory. Br J Soc Clin Psychol.

[27] Moyers T.B., Martin T., Manuel J.K., Miller W.R., Ernst D. (2007).

[28] Moyers T.B., Manuel J.K., Ernst D. (2014).

[29] Svendsen O.L., Haarbo J., Hassager C., Christiansen C. (1993). Accuracy of measurements of body composition by dual-energy x-ray absorptiometry in vivo. Am J Clin Nutr.

[30] Visser M., Fuerst T., Lang T. (1999). Validity of fan-beam dual-energy X-ray absorptiometry for measuring fat-free mass and leg muscle mass. J Appl Physiol.

[31] Hind K., Oldroyd B., Truscott J.G. (2011). In vivo precision of the GE Lunar iDXA densitometer for the measurement of total body composition and fat distribution in adults. Eur J Clin Nutr.

[32] Rezzi S., Ginty F., Beaumont (2009). Body composition precision with the Lunar iDXA. J Clin Densitom.

[33] Rothney M.P., Martin F.P., Xia Y. (2012). Precision of GE Lunar iDXA for the measurement of total and regional body composition in nonobese adults. J Clin Densitom.

[34] Markland D., Tobin V. (2004). A modification to the behavioural regulation in exercise questionnaire to include an assessment of amotivation. J Sport Exerc Psychol.

[35] Ryan R.M., Deci E.L. (2000). Self-determination theory and the facilitation of intrinsic motivation, social development, and well-being. Am Psychol.

[36] Cid L., Monteiro D., Teixeira D. (2018). The behavioral regulation in exercise questionnaire (BREQ-3) Portuguese-version: evidence of reliability, validity and invariance across gender. Front Times.

[37] Wilson P.M., Bengoechea E.G. (2010). The relatedness to others in physical activity scale: evidence for structural and criterion validity. J Appl Biobehav Res.

[38] Vlachopoulos S.P., Michailidou S. (2006). Development and initial validation of a measure of autonomy, competence, and relatedness in exercise: the Basic Psychological Needs in Exercise Scale. Meas Phys Educ Exerc Sci.

[39] Schunk D.H., Zimmerman B.J. (2012).

[40] Vlachopoulos S.P., Ntoumanis N., Smith A.L. (2010). The basic psychological needs in exercise scale: translation and evidence for cross‐cultural validity. Int J Sport Exerc Psychol.

[41] Keppel G., Wickens T.D. (2004).

[42] Strunk K.K., Mwavita M. (2020).

[43] Faul F., Erdfelder E., Lang A.G., Buchner A. (2007). G∗ Power 3: a flexible statistical power analysis program for the social, behavioral, and biomedical sciences. Behav Res Methods.

[44] Mustedanagić J., Bratić M., Milanović Z., Pantelić S.D. (2016).

[45] Kim S., Han G. (2016). Effect of a 12-week complex training on the body composition and cardiorespiratory system of female college students. J Phys Ther Sci.

[46] Lee D.H., Keum N., Hu F.B. (2018). Predicted lean body mass, fat mass, and all cause and cause specific mortality in men: prospective US cohort study. BMJ.

[47] Gourlan M., Sarrazin P., Trouilloud D. (2013). Motivational interviewing as a way to promote physical activity in obese adolescents: a randomised-controlled trial using self-determination theory as an explanatory framework. Psychol Health.

[48] Friederichs S.A., Oenema A., Bolman C., Lechner L. (2015). Long term effects of self-determination theory and motivational interviewing in a web-based physical activity intervention: randomized controlled trial. IJBNPA.

[49] Webber K.H., Tate D.F., Quintiliani L.M. (2008). Motivational interviewing in internet groups: a pilot study for weight loss. J Am Diet Assoc.

[50] West D.S., Gorin A.A., Subak L.L. (2011). A motivation-focused weight loss maintenance program is an effective alternative to a skill-based approach. Int J Obes.

[51] Silva M.N., Vieira P.N., Coutinho S.R. (2010). Using self-determination theory to promote physical activity and weight control: a randomized controlled trial in women. J Behav Med.

[52] Teixeira P.J., Carraça E.V., Markland D., Silva M.N., Ryan R.M. (2012). Exercise, physical activity, and self-determination theory: a systematic review. IJBNPA.

[53] Williams G.C., Grow V.M., Freedman Z.R., Ryan R.M., Deci E.L. (1996). Motivational predictors of weight loss and weight-loss maintenance. J Pers Soc Psychol.

[54] Al-Musharaf S., Aljuraiban G., Bogis R. (2021). Lifestyle changes associated with COVID-19 quarantine among young Saudi women: a prospective study. PLoS One.

[55] Dragun R., Vecek N.N., Marendi C.M. (2021). Have lifestyle habits and psychological well-being changed among adolescents and medical students due to COVID-19 lockdown in Croatia?. Nutrients.

[56] Palmer K., Bschaden A., Stroebele-Benschop N. (2021). Changes in lifestyle, diet, and body weight during the first COVID 19 ‘lockdown’in a student sample. Appetite.

[57] Stanila A.M., Oravitan M., Matichescu M.L. (2021). Factors predisposing to weight gain in young adults during COVID19 home confinement. Timisoara Phys Educ Rehabil J.

[58] Kim M.H., Yeon J.Y. (2021). Change of dietary habits and the use of home meal replacement and delivered foods due to COVID-19 among college students in Chungcheong province. Korean J Nutr.

[59] Chaturvedi K., Vishwakarma D.K., Singh N. (2021). COVID-19 and its impact on education, social life and mental health of students: a survey. Child Youth Serv Rev.

[60] da Mota Santana J., Milagres M.P., Dos Santos C.S. (2021). Dietary intake of university students during COVID-19 social distancing in the Northeast of Brazil and associated factors. Appetite.

[61] Tan S.T., Tan C.X., Tan S.S. (2021). Physical activity, sedentary behavior, and weight status of university students during the COVID-19 lockdown: a cross-national comparative study. Int J Environ Res Publ Health.

[62] Baquerizo-Sedano L., Chaquila J.A., Aguilar L. (2021). AntiCOVID-19 measures threaten our healthy body weight: changes in sleep and external synchronizers of circadian clocks during confinement. Clin Nutr.

[63] Brancaccio M., Mennitti C., Gentile A. (2021). Effects of the COVID-19 pandemic on job activity, dietary behaviours and physical activity habits of university population of Naples, Federico II-Italy. Int J Environ Res Publ Health.

